# Emotion Detection Based on Pupil Variation

**DOI:** 10.3390/healthcare11030322

**Published:** 2023-01-21

**Authors:** Ching-Long Lee, Wen Pei, Yu-Cheng Lin, Anders Granmo, Kang-Hung Liu

**Affiliations:** 1 Ph.D. Program of Management, Chung Hua University, Hsinchu 300, Taiwan; 2 Department of Business Administration, Chung Hua University, Hsinchu 300, Taiwan; 3 Department of Mechanical and Computer-Aided Engineering, Overseas Chinese University, Taichung 407, Taiwan

**Keywords:** affective computing, emotional recognition, pupillary response, machine learning

## Abstract

Emotion detection is a fundamental component in the field of Affective Computing. Proper recognition of emotions can be useful in improving the interaction between humans and machines, for instance, with regard to designing effective user interfaces. This study aims to understand the relationship between emotion and pupil dilation. The Tobii Pro X3-120 eye tracker was used to collect pupillary responses from 30 participants exposed to content designed to evoke specific emotions. Six different video scenarios were selected and presented to participants, whose pupillary responses were measured while watching the material. In total, 16 data features (8 features per eye) were extracted from the pupillary response distribution during content exposure. Through logistical regression, a maximum of 76% classification accuracy was obtained through the measurement of pupillary response in predicting emotions classified as fear, anger, or surprise. Further research is required to precisely calculate pupil size variations in relation to emotionally evocative input in affective computing applications.

## 1. Introduction

Increasingly, machine learning and artificial intelligence technologies and techniques are being used in the field of affective computing in order to detect human emotion [1,2]. Emotion detection has wide applications in several fields, including healthcare, psychology, business, human-centered computing, ambient intelligence, and interactive design. In this study, emotions are defined as mental and/or emotional states that arise spontaneously and last anywhere from a few seconds to a few minutes. This stands in contrast to moods, which can last for hours, or even days or months. Certain emotions are typically classified as general and basic evolutionary survival-based traits, such as fear and disgust. Another category includes specific and complex emotions, which are, to a larger extent, learned by social developmental processes and individual experiences, such as jealousy, embarrassment, gregariousness, etc. This latter category is less directly connected with survival and has a more specific purpose of social adaptation. Ekman was one of the pioneers who systematically defined human emotions and proposed a discrete emotional model using six universal emotions: happiness, surprise, anger, disgust, sadness, and fear [3]. Some psychologists propose a simpler model to depict emotional status across dimensions: valence, arousal, and dominance [4]. Valence is the negative–positive valence of emotion and varies from unpleasant to pleasant. Arousal is the intensity of emotion and changes from inactive (e.g., boredom) to active (e.g., excitement). Dominance defines and distinguishes whether an emotion is dominant (e.g., anger) or submissive (e.g., fear).

Currently, many emotion recognition systems exist that are able to detect, process, and classify the emotional states of a human subject. Most of them operate by obtaining various physiological signals, facial expressions, and voice under exposure to specific stimuli, with the purpose of recognizing specific emotions [5,6,7,8,9,10]. Physiological signals, such as respiration, heart rate, skin temperature, and pupillary response, which are subconsciously regulated by the autonomic nervous system (ANS), are considered to be among the most significant signals of various emotional states. These physiological signals, along with cardiac functions (heart rate and blood volume pulse), muscle tension (electrical activity), skin conductance, and electrical activity of the brain, can be captured and recorded under different emotional circumstances. One feature of pupillary response, pupil dilation, is recognized as an indicator of emotion (especially arousal) and can, through data processing, be used for creating models designed for the automatic recognition of emotion [11,12,13,14,15]. Recently, eye tracking systems have been added as a key function on smart glasses; an increased amount of information collected from the human eye is therefore to be expected in the future.

As sensor technology advances, pupil variances and eye movement during emotional events can be measured with an increasing degree of accuracy. This is largely conducted by using desktop-based or wearable eye trackers, which are devices for measuring an individual’s eye focus, eye positions, and eye movements during emotionally relevant events [15,16,17]. The camera monitors light source reflection along with visible eye features such as pupillary response in terms of pupil diameter changes, as well as other information such as blinking frequency. Previous studies have discussed the relationship between sentiment changes and blink frequency, pupil diameter, and eye movement (saccade, fixation, pursuit, and so on). Pupil diameter changes can be observed during positive or negative sentiments, and fixation duration is related to parameters such as attention and valence [14]. Relevant eye features from stimuli are classified by machine learning algorithms or other classifiers to correctly recognize emotions. Tarnowski et al. [14] applied a support vector machine (SVM) classifier to detect three emotional states, namely (1) high arousal and high valence, (2) low arousal and moderate valence, and (2) high arousal and low valence), while study participants watched 21 video clips. Eye movement data were collected, and 18 data features (9 per eye) were extracted across the categories of fixation, fixation duration, saccade amplitude, saccade duration, and pupil diameter. A maximum classification accuracy of 80% was achieved in recognizing the three abovementioned classes of emotions, implying that emotion classification can be successfully used in practice [14]. Lu et al. (2015) investigated 16 eye movements and identified the intrinsic patterns of these eye movements corresponding to three emotional states, namely (1) positive, (2) neutral, and (3) negative. It revealed that the best accuracy (87.59%) was achieved through a fuzzy integral fusion strategy, whereas the accuracies of solely using eye movements and electroencephalogram (EEG) data were 77.80% and 78.51%, respectively [17]. Moreover, multimodal emotion recognition based on EEG signals and eye-tracking data were obtained to build a fusion model with the purpose of improving the performance of emotion recognition. Two fusion strategies—feature level fusion and decision level fusion—were adopted to build classifier models, which approached respective maximum classification accuracies of 73.59% and 72.98% in recognizing the three abovementioned sentiment categories [18].

Some studies indicate that pupil diameter is modulated by emotional arousal related to the extent of mental effort required to regulate automatic emotional responses [18,19]. Several studies have demonstrated that emotional arousal during affective visual or auditory emotional stimulation is associated with pupillary response [18,20,21]. While neutral and positive images are mostly characterized by a fast contraction followed by a sustained dilatation under the baseline, negative images tend to trigger a slower and smaller contraction, followed by a large, sustained dilatation that is maintained during image exposure [22,23,24].

Most previous studies utilize multimodal measurements to detect positive, negative, and neutral emotions and specific emotional statuses such as happiness, sadness, surprise, fear, or disgust [6]. Dzedzickis et al. reviewed the sensors and methods for emotion recognition and indicated that the reliability, precision, and speed of emotion evaluation depend not only on the used sensors and methodology but also on the applied signal processing and analysis technique [10]. They stated that measurements based on unconscious responses and data collected directly from body electrical signals (such as electrooculography, electromyogram, electrocardiography, etc.) usually provide more reliable and precise results for emotion recognition. However, measurements based on non-electrical parameters tend to suffer from latency and inadequate accuracy. However, their main advantage is the potential fit for field applications and for approximate emotional state evaluations. Therefore, while more and more studies attempt to recognize more complex emotions by applying contact and wearable sensors to classify emotions, only a very limited number of studies have developed effective data extraction of features of pupillary response controlled by ANS for emotion recognition. Therefore, this study aims to understand the relationship between emotion and pupil dilation and attempts to recognize emotion by pupil variance during the video exposure.

## 2. Methodology

### 2.1. Participants

Thirty volunteer participants (gender-equaled) were recruited to participate in this study. Their average age was 21.1 (SD = 0.26, range between 20 and 24) years old. All participants were college students and self-reported normal auditory and visual capacity and absence of any physical and/or mental illness. Prior to the experiment, participants were asked to check that they had never watched the material of emotional stimulus used in the experiment. All procedures performed in the study involving human participants were conducted in accordance with relevant ethical standards. The procedures conformed to the Declaration of Helsinki and were approved by the ethics committee of the Institutional Review Board (IRB) at the National Tsing Hua University of Taiwan.

### 2.2. Scenario and Emotion Evoking

According to Ekman’s emotion theory, the six primary human emotions comprise happiness, sadness, fear, disgust, anger, and surprise [25,26,27]. The definition of each emotion is listed as follows:

Happiness: pleasant feelings of contentment, joy, gratification, satisfaction, and well-being. Happiness is generally associated with smiling, laughing, and relaxed facial expressions.

Sadness: feelings of upset, disappointment, grief, hopelessness, disinterest, and dampened mood. Sadness is generally accompanied by crying, tears, frowning, withdrawal from social interactions, etc. 

Fear: one of the primary emotions, which generates the fight-or-flight response to keep safe from danger and threat. In general, it is considered a negative emotional state; however, fear is a survival mechanism for safety and health.

Disgust: another primary emotion that plays a crucial role in survival. It refers to the feelings of repulsion and avoidance of substances that can be dangerous to health; disgust serves as an evolutionary and adaptive trait to reject any substance that could be contaminated or toxic.

Anger: an intense, unpleasant feeling that often prompts one to act without thinking. Anger is one of the most evolutionary beneficial emotions among the 6 basic emotions. It enables humans to increase physiological responses. Among other things, anger can increase cardiac activity, enhance muscle tension, and augment breathing rate.

Surprise: a reaction to something unexpected and unprecedented, a sense of astonishment, wonder, or amazement. Surprise can be positive or negative, based on the subject’s experience, expectations, and environment.

According to the above description and definitions, 18 videos comprising content classified across the six emotions were extracted from YouTube and presented randomly to 10 subjects in the pilot run in order to test whether these videos evoked the intended emotions. After exposure to the material, pilot participants were asked to rate emotions evoked on a scale from 0 (not at all) to 10 (extremely strong). The highest average scores (≥7 points) of each emotional scenario were selected as the material of emotional stimulus in subsequent experimental procedure. Six scenarios (videos) were selected and presented to participants in order to induce the six emotions. The content of the six video clips was simply depicted as follows:

Happiness was evoked by a series of amusing, jocular, and humorous scenarios.

Sadness was induced by a story about the relationship between grandmother and grandchild. As the grandmother gets older, she develops dementia. The video is full of sorrow and melancholy.

Fear was evoked by a horror and suspense film; the story involves evil zombies in an old castle.

Disgust was induced through a short film that presented zoom-in shots of putrid acne squeezing and festering ulcers accompanied by other uncomfortable images.

Anger was provoked through a short film that displays an unfair situation in which a teacher humiliates students by using verbal insults and physical violence in a public setting.

Surprise was produced through a clip that shows a series of unexpected and unpredictable magic tricks.

Each video clip lasted for approximately 3–5 minutes. Before each video clip presentation, there were three minutes of idle intervals for participants to take a break and return to a baseline emotional state.

### 2.3. Experiemental Setting

**Figure 1:**
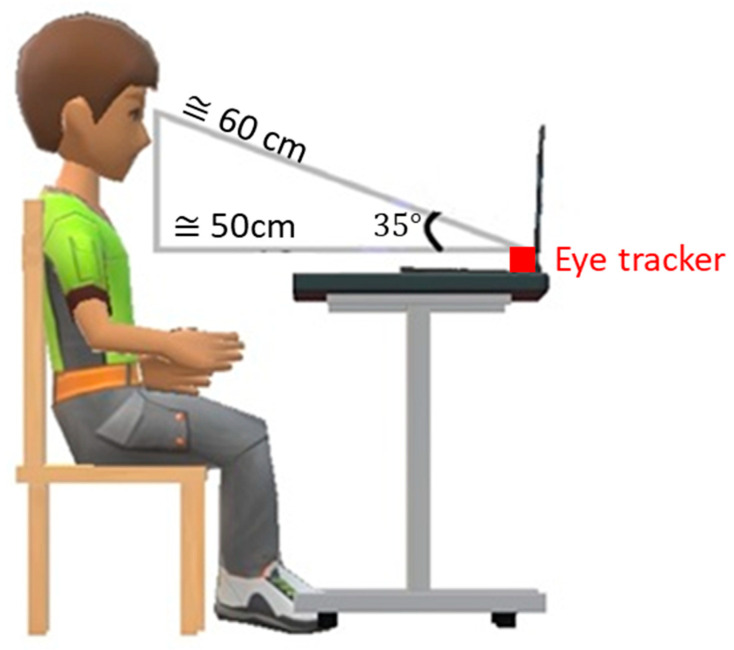
Illustration of the experimental setting.

Participants were invited to sit in an adjustable chair to make themselves comfortable at the experimental desk, at which there was a standard 1920 × 1080 p computer monitor. For optimal tracking, the eye tracker was placed in such a way so that the gaze angle (α) would not exceed 36° when the participant is located about 65 cm (26″) from the eye tracker. The allowable operating distance (from eye tracker to participant’s eyes) for the Tobii Pro X3-120 Eye Tracker is 50–90 cm (19.6–35.4″). In this study, as Figure 1 illustrates, the distance from the participant’s eyes to the eye tracker and monitor was approximately 60 cm, and the gaze angle (α) was around 35°.

Participant head movements would be performed unconsciously during the experiment, which could have an impact on data quality. In order to make sure that pupillary response data could be tracked properly, each participant must have at least one eye within the trackable area at all times. In order to avoid environmental auditory interruption, participants were equipped with standard headphones, which provided a suitable sound level with a consistent sound stimulus throughout the experimental procedure. The indoor temperature was controlled to 27 °C.

### 2.4. Measurements and Data Processing

**Figure 2:**
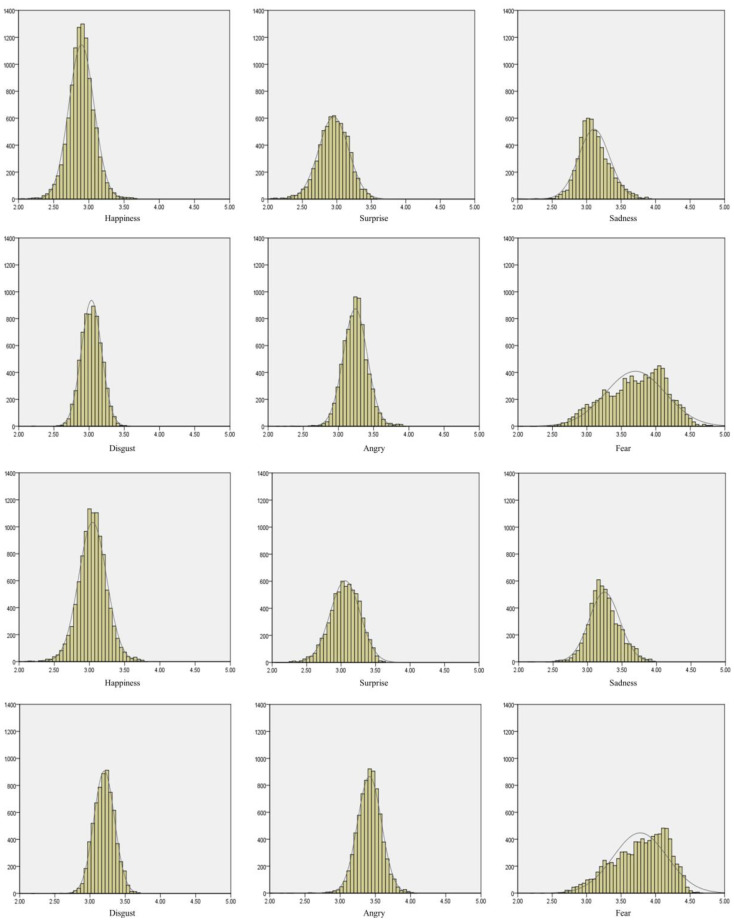
Six emotions and pupil dilation recording for both eyes. (The top six graphs are for left pupil; the bottom six graphs are for right eye. X-axis is pupil dilation/mm; Y-axis is count).

An infrared camera-based eye tracking system was applied to collect pupillary response data with a sampling rate of 40 Hz during the experimental procedure, meaning that pupillary response is measured 40 times per second or at a rate of 2400 data points per minute. The pupil diameter of both eyes was recorded throughout the video exposure process. Total data points collected per exposure approximates between 7000 and 12,000, depending on the quality of data collection for each participant and the length of the respective videos. A certain extent of data loss during recording is unavoidable due to participants blinking or moving their heads. Figure 2 illustrates the left and right pupillary response distribution for each of the six videos. It reveals that although the respective data from the left and right pupils were unequal, both pupils displayed similar data distribution tendencies.

For data processing, normal distribution testing was initially conducted using IBM’s SPSS Statistics. Then, one-way analysis of variance (ANOVA) was utilized to analyze the effect between these emotions. Duncan’s multiple range test (DMRT) was applied as a post hoc test to measure specific differences between these emotions. The significance level was set at a *p*-value of 0.01.

### 2.5. Feature Selection and Classification Model

The steps associated with emotion recognition and its cognate classification systems comprise signal acquisition, data preprocessing, dataset construction, feature extraction, and model building. Following previous studies analyzing physiological signals [17], 16 extracted data features (8 per eye) include minimum value, maximum value, first quartile (q1), median value (q2), third quartile (q3), mean value, standard deviation (SD), and variance of pupillary response data extracted during exposure. The values of these features are based on the frequency distribution of pupil size during emotional exposure. The features are defined as follows:

Minimum value: minimal pupil diameter value.

Maximum value: the largest pupil diameter value.

q1: the 25th percentile pupil diameter value.

Median value or q2: the 50th percentile pupil diameter value.

q3: the 75th percentile pupil diameter value.

Mean value: the average value of pupillary response (diameter).

SD: the standard deviation between values.

Variance: the maximum value minus the minimum pupil diameter value.

Four classification models, comprising K-nearest neighbor (KNN), decision tree (DT), random forest (RF), and logistic regression (LR), were used to build the prediction model [6,10]. The model’s algorithms were implemented using Orange Data Mining Library (Orange. Classification Tree: Orange Data Mining Library 3 Documentation, n.d.) [28]. In this study, multiple DT models were created using many different combinations of records and features. Orange was chosen due to the simplicity of generating multiple models using different variables when compared to generating them manually in Python. The software also allows for the filtering of records, enabling the creation and manipulation of models by using specific records as inputs.

Further, the study selected performance evaluation parameters, which are used to examine the efficiency of classifiers. In general, the receiver operating characteristic (ROC) curve considers the number/probability of the events of true positives (TPs), true negatives (TNs), false positives (FPs), and false negatives (FNs). They are under the ROC curve; AUC was used to detect the ability of the classifier model. The accuracy is then measured via an index to compute the probability of the number of TPs and TNs in the total observations (the events of positives and negatives is an index to measure how the overall model performs) [6,10].

(1) $$\mathrm{Accuracy}=\frac{\left(\mathrm{TP}+\mathrm{TN}\right)}{\left(\mathrm{TP}+\mathrm{TN}+\mathrm{FP}+\mathrm{FN}\right)}$$

Recall measures the percentage of TP cases that are correctly identified. Precision measures the cases predicted to be positive and indicates the percentage of them are TPs. Considering the conflicting feature between precision and recall, the F1 Score is created to have a balanced metric between recall and precision values.

(2) $$\mathrm{Recall}=\frac{\mathrm{TP}}{\left(\mathrm{TP}+\mathrm{FN}\right)}$$

(3) $$\mathrm{Precision}=\frac{\mathrm{TP}}{\left(\mathrm{TP}+\mathrm{FP}\right)}$$

(4) $$F1=2\times \frac{\mathrm{Precision}\times \mathrm{Recall}}{\mathrm{Precision}+\mathrm{Recall}}$$

The confusion metric is a performance measurement for a machine learning algorithm and is a summarized table of the number of correct and incorrect predictions (or actual and predicted values) yielded by a classifier (or classification model) for binary classification tasks. It could be presented in the form of a square matrix where the column represents the actual values, and the row depicts the predicted value of the model and vice versa. A satisfactory classifier will have large values across the diagonal and small values of the diagonal in the square matrix. It also provides better insight in particulars to the classification model with regard to the correctness and what types of errors are being created.

## 3. Results

As suggested with regard to signal collection, some of the participants’ pupillary response information tends to be lost due to blinking and head/posture movement. In practice, it is therefore normal to collect unequal pupil dimensions for either eye during measurement. As seen in Figure 2, there were 10,378 pupil data collected for the left eye and 10,303 pupil data collected for the right eye during one participant’s exposure to the happiness scenario video. Pupil diameter means were 2.89 (SD = 0.18) and 3.04 (SD = 0.19) millimeters for the left and right eye under this emotion, respectively. However, from the frequency distribution of the happiness scenario, data for both eyes have similar patterns when exploring the same emotion. Similarly, unbalanced pupil data collection appeared in all emotional scenarios; this is also partly due to the variation in film length, which ranged between three to five minutes. In spite of some data loss, we can still clearly observe that there were similar frequency distribution patterns between both eyes.

For data processing, a total of 360 frequency distributions (6 emotions × 30 subjects × 2 eyes) of pupillary response were plotted into our main diagram. The normal distributions of each emotion were tested with the SPSS statistics package. As seen in Table 1, the normality of pupillary response data was assessed using Kolmogorov–Smirnov (K–S), indicating that the frequency distribution of pupil dilation for the six emotions could be considered a normal distribution in features of minimum, maximum, q1, q2, q3, and mean values. Only pupillary response frequency distribution in features of standard deviation and variance is a non-normal distribution.

**Figure 3:**
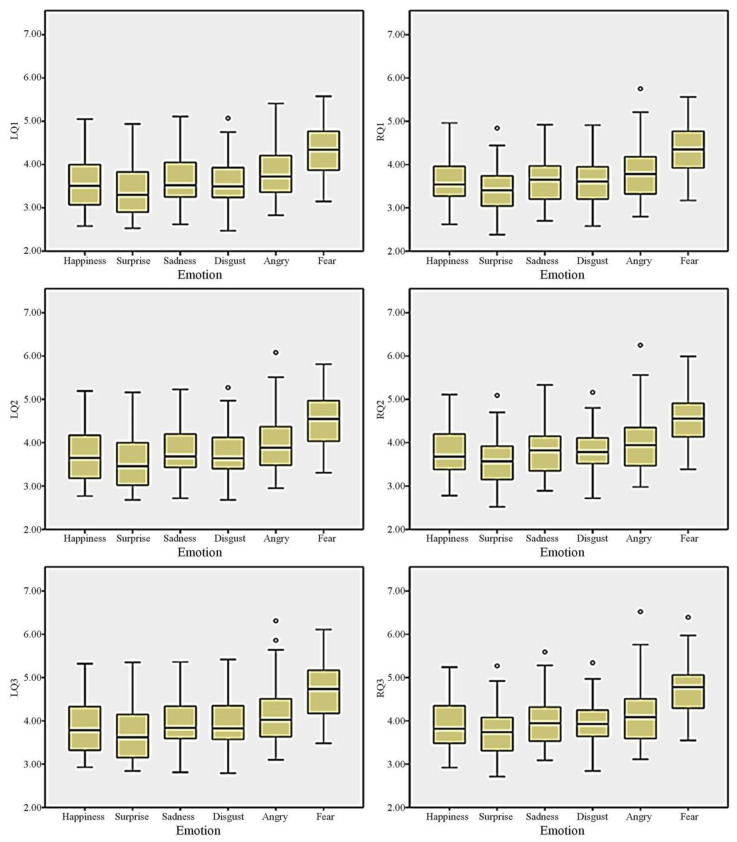
The averaged values of q1, q2 , and q3 for both eyes between six emotions. The cycles represent outliers, which are lower than q1 − 1.5 × interquartile range or higher than q3 + 1.5 × interquartile range.

Based on the above data characteristics, to further illustrate and analyze the emotional distribution while watching the videos, a total of 12 features (excluding standard deviation and variance of pupil dilation of both eyes) were selected and extracted during each individual’s emotional exposure. ANOVA was used to analyze the effect between these emotions. DMRT was applied as a post hoc test to measure specific differences. Table 2 displays descriptive statistics, including means and standard deviation for each feature. Figure 3 illustrates the averaged values of q1, q2, and q3—it displays a tendency that the fear emotion tends to have a greater pupil diameter; conversely, the surprise sentiment has a lower pupil diameter.

Table 3 reveals the results of the analysis of variances among the six emotions. Except for the parameters of standard deviation and variance between both pupils, most other parameters are significantly different for each individual emotion (*p* < 0.01). This indicates a significant difference between the six emotions with regard to the minimum value, maximum value, quartiles, and mean value extracted from individual frequency distributions (*p* < 0.01). The post hoc DMRT was applied to measure specific differences between these emotions. The consistent tendency of Duncan’s multiple range test found in Table 3 suggests that the fear sentiment displays greater pupil diameter between its minimum value, maximum value, quartiles, and mean value than any other emotion; conversely, the surprise sentiment displays the lowest pupil diameter variation between the extremes of its values.

## 4. Discussions

In general, an emotion or current feeling evoked by a given event is spontaneous and only lasts for a few seconds or minutes. Specifics of emotional adaptation and regulation vary between individuals according to their developmental state, environment, and social experience. For example, some people enjoy watching horror films and might have more effective sentiment regulation skills than people who avoid such films in anticipation of an uncomfortable degree of fear. Thus, interindividual differences exist in emotion regulation. In this study, the emotions of sadness, disgust, and happiness did not have significant differences in the selected features with regard to their respective frequency distribution. One possible explanation is that these three emotions evoke similar arousal states in spite of the fact that some are negative (sadness and disgust) and another is positive (happiness) [14,15]. The more noteworthy observation of this study is the remarkable differences in pupillary response between fear, anger, and surprise. It is unsurprising that fear causes greater pupil diameter because of its association with higher arousal; these findings are congruent with those of previous studies [7,18,19,22,24]. The video designed to evoke surprise seems to have brought relaxed, satisfied, and positive feelings to the participants, as evidenced by smaller pupillary response variation than other measured emotions. However, some studies have claimed that pupil size caused by anger-inducing stimuli was larger than the pupil size caused by fear-inducing stimuli [29,30]. The pupillary response is moderated by the ANS and regulated subconsciously. Both anger- and fear-inducing stimuli may cause greater pupil sizes because of their associations with negative valence and intense arousal; differences may be caused by the intensity of the stimulus. The video designed to evoke fear seems to have a higher intensity than the video designed to evoke anger. Future studies should consider increasing the diversity of the videos to balance this effect.

Recently, some studies have explored the relationship between emotions and pupil size. Most of them adopted images as emotional stimuli and collected the immediate pupil response within a few seconds of exposure. [29,30] This study lengthened the time period to identify and visualize the features of pupillary response distribution (Figure 2) among each of the six emotion stimuli. Based on the results, there were several identifiable features that distinguish emotions. For example, the basic statistics features (minimum, maximum, q1, q2, q3, and mean) have significant variation across emotions. The pupillary response distribution of fear had a negative skew, which could be directly reflected in the features of descriptive statistics (q2 > mean, as shown in Table 2). Hence, this research applies machine learning algorithms to develop emotion recognition and classification systems through statistical features of pupil dilation.

The 16 selected features were used as input data for classification. As described above, four classification models were utilized in order to build the prediction model, namely KNN, DT, RF, and LR [6,10]. KNN identifies from its neighbors similar points in the training data that are closest to the test observation and classifies them by estimating the conditional probability of belonging to each class and choosing the class with the largest probability. DT uses a tree-like graph to predict the value of a target variable by learning simple decision rules inferred from the data features. RF is an ensemble learning technique consisting of the aggregation of a large number of decision trees, resulting in a reduction of variance compared to single DTs. LR is a regression analysis technique. Regression analysis is a set of statistical processes that can be used to estimate relationships among variables. Predicating a qualitative response for an observation can be referred to as classifying that observation since it involves assigning the observation to a category or class. LR is an instance of a classification technique that can be used to predict a qualitative outcome.

Emotion recognition and classification systems consist of signal acquisition, data preprocessing, dataset construction, feature extraction, and model building. Following previous analyses of physiological signals [24,31], 16 features were selected and extracted during each individual’s exposure to produce pupillary response samples. With regards to the classification model of the six emotions, it displays an unsatisfactory prediction ability to detect individual emotions by pupil diameter distribution. The AUC, which was used to detect the ability of these four classifier models, was between 0.77 and 0.89, with an accuracy rating of 0.45–0.57. One explanatory cause is that pupillary response distributions were plotted in a certain range (2–6 mm), and some emotional responses could not be observed and measured using pupil size variation as a single variable—analyzing additional features is required for precise collection in order to build a robust prediction model in the future. Moreover, based on the results illustrated in Table 4, pupil features from the emotions of fear, anger, and surprise have significant differences. Therefore, the classifier was applied further to predict these three emotions. Classification accuracies achieved for these three emotions are shown in Table 4. The highest accuracy of 76% for the classification of these three emotions was obtained using the LR classifier. The precision (P), recall (R), and F1-score (F1) were calculated for these three classes of emotions as well. Table 5 presents the confusion matrix on the LR classifier for fear, anger, and surprise. It implies that for the prediction of surprise, the LR model has a satisfactory prediction ability. However, for fear and anger, the correct prediction rate of only 60% is insufficient. One explanation for this may be that the content in the anger and fear video clips both comprise physical violence and bloodiness, which both may induce irritable, anxious, and uncomfortable feelings.

Previous studies have detected positive, negative, and neutral emotions with ML techniques, focusing on affective valence and arousal levels. It is reasonable to surmise that emotional evocation is situational and event-based and that it varies by individual experiences and personal preferences, which, inter alia, is related to sociocultural developmental processes. On the other hand, the pupillary response is moderated by the ANS and regulated subconsciously and therefore is not entirely subject to influences from social learning processes with regard to affective changes. Many studies suggest that pupil diameter is modulated by arousal status because a higher level of mental effort is required to respond proportionally to outside stimuli. A similar tendency was observed in this study—the sentiment of fear predicts greater pupillary response owing to its tendency to externally produce feelings of tension, anxiety, and pressure. Conversely, feelings of surprise caused by positive, amusing, and relaxed stimuli produce less pupillary response.

## 5. Conclusions

Past studies have tended to apply the data of pupil size within a very short period of the during and after exposure to stimuli. However, durations of real-world exposure to stimuli are unpredictable. Moreover, emotional states could last from seconds to a few minutes. Hence, this research tries to adopt a longer time period to identify and visualize the difference in pupillary response distribution among each of the six emotion-evoking stimuli. Response to primary emotions such as fear, anger, and surprise may have less potential for interference by other factors as they revealed a consistent pupil frequency distribution for both eyes in this study.

Applying machine learning to learn and build models for specific emotional predictions is possible. In this study, four classifier models displayed unsatisfactory prediction abilities in detecting six emotions by pupil diameter distribution—the accuracy is limited to 0.45–0.57. However, for detecting the three primary emotions (fear, anger, and surprise), the highest accuracy of 76% predictive ability was achieved by an LR classifier. It implies that proper extraction of more specific features, improved population sampling, and high-quality quality big data collection from certain environments are research variables required for deeper and more successful and precise affective detection.

## 6. Limitation and Future Research

Emotional evocation could be contextual and event-based—the same stimulus might cause different reactions across different individuals based on their particular experiences and emotional states. This means that exposure to the same content will result in variation observed between individuals. Moreover, the somewhat narrow demographic participants taking part in this experiment (young adults) may have compounded this effect and affected the result. Conversely, it is possible that, in such a coherent age group of subjects, emotions were evoked more precisely. Therefore, it should be kept in mind that accurate comparisons are difficult because each study uses a different way of evoking emotions, a different set of eye-tracking features, and combinations of scholarly teams. The sample of this study was limited and recruited only young adults from one country, which could limit generalizability. To develop a classification model that can work in practice, extended data collection with diversity is necessary in the future.

## Figures and Tables

**Table 1:** Normality test for all features.

Features		Happiness	Surprise	Sadness	Disgust	Anger	Fear
Left	Minimum	0.08	0.20	0.16	0.20	0.20	0.20
	Maximum	0.20	0.14	0.20	0.20	0.20	0.20
	q1	0.20	0.20	0.19	0.20	0.07	0.20
	q2	0.20	0.20	0.20	0.20	0.20	0.20
	q3	0.20	0.20	0.20	0.20	0.20	0.20
	Mean	0.20	0.20	0.19	0.20	0.20	0.20
	SD	0.00 *	0.10	0.01 *	0.00 *	0.00 *	0.00 *
	Variance	0.20	0.20	0.09	0.00 *	0.03 *	0.20
Right	Minimum	0.20	0.20	0.20	0.20	0.20	0.20
	Maximum	0.20	0.20	0.20	0.16	0.19	0.20
	q1	0.20	0.11	0.20	0.20	0.20	0.20
	q2	0.20	0.09	0.20	0.20	0.07	0.20
	q3	0.20	0.05	0.20	0.20	0.14	0.20
	Mean	0.20	0.13	0.20	0.20	0.20	0.20
	SD	0.00 *	0.09	0.00 *	0.00 *	0.00 *	0.02 *
	Variance	0.11	0.04 *	0.08	0.03 *	0.03 *	0.06

* The significance level was at *p* < 0.05.

**Table 2:** Averaged values and standard deviations for 16 features (8 per eye).

Pupil (mm) Features		Happiness	Surprise	Sadness	Disgust	Anger	Fear
Left	Minimum	2.25	2.18	2.36	2.38	2.6	2.67
		(0.49)	(0.53)	(0.48)	(0.52)	(0.52)	(0.54)
	Maximum	4.5	4.45	4.66	4.61	4.76	5.32
		(0.69)	(0.69)	(0.73)	(0.77)	(0.77)	(0.66)
	q1	3.59	3.4	3.62	3.57	3.82	4.31
		(0.62)	(0.61)	(0.6)	(0.59)	(0.68)	(0.62)
	q2	3.74	3.56	3.78	3.75	3.99	4.51
		(0.63)	(0.61)	(0.61)	(0.61)	(0.74)	(0.64)
	q3	3.88	3.72	3.95	3.91	4.14	4.69
		(0.64)	(0.63)	(0.61)	(0.63)	(0.77)	(0.65)
	Mean	3.72	3.55	3.79	3.74	3.96	4.48
		(0.62)	(0.62)	(0.6)	(0.59)	(0.69)	(0.62)
	Standard deviation	0.24	0.27	0.2	0.28	0.28	0.3
		(0.07)	(0.06)	(0.11)	(0.12)	(0.17)	(0.09)
	Variance	2.25	2.27	2.3	2.23	2.17	2.65
		(0.54)	(0.55)	(0.58)	(0.61)	(0.7)	(0.58)
Right	Minimum	2.28	2.23	2.35	2.28	2.5	2.72
		(0.43)	(0.51)	(0.37)	(0.44)	(0.48)	(0.52)
	Maximum	4.65	4.57	4.79	4.7	4.86	5.4
		(0.75)	(0.73)	(0.68)	(0.69)	(0.88)	(0.72)
	q1	3.62	3.42	3.64	3.58	3.85	4.32
		(0.57)	(0.58)	(0.57)	(0.53)	(0.68)	(0.6)
	q2	3.77	3.58	3.83	3.77	4.03	4.54
		(0.59)	(0.6)	(0.6)	(0.55)	(0.76)	(0.64)
	q3	3.91	3.74	4	3.94	4.19	4.74
		(0.61)	(0.62)	(0.61)	(0.58)	(0.78)	(0.65)
	Mean	3.76	3.57	3.82	3.76	4	4.51
		(0.58)	(0.58)	(0.57)	(0.53)	(0.69)	(0.61)
	Standard deviation	0.25	0.27	0.3	0.3	0.29	0.32
		(0.09)	(0.08)	(0.14)	(0.17)	(0.19)	(0.13)
	Variance	2.37	2.34	2.43	2.42	2.36	2.68
		(0.71)	(0.75)	(0.62)	(0.73)	(0.73)	(0.66)

The significance level was at *p* < 0.05.

**Table 3:** ANOVA results and post hoc testing.

Pupil	Features	F	Sig.	Duncan’s Multiple Range Test
Left	Minimum	4.175	0.00 *	Fear, anger > anger, disgust, sadness > disgust, sadness, happiness, surprise
	Maximum	5.753	0.00 *	Fear > anger, sadness, disgust, happiness, surprise
	q1	7.947	0.00 *	Fear > anger, sadness, happiness, disgust > sadness, happiness, disgust, surprise
	q2	8.038	0.00 *	Fear > anger, sadness, disgust, happiness > sadness, disgust, happiness, surprise
	q3	8.058	0.00 *	Fear > anger, sadness, disgust, happiness > sadness, disgust, happiness, surprise
	Mean	8.189	0.00 *	Fear > anger, sadness, disgust, happiness > sadness, disgust, happiness, surprise
Right	Minimum	4.843	0.00 *	Fear, anger > anger, sadness, disgust, happiness > sadness, disgust, happiness, surprise
	Maximum	4.767	0.00 *	Fear > anger, sadness, disgust, happiness, surprise
	q1	8.575	0.00 *	Fear > anger, sadness, happiness, disgust > sadness, happiness, disgust, surprise
	q2	8.593	0.00 *	Fear > anger, sadness, happiness, disgust > sadness, happiness, disgust, surprise
	q3	8.857	0.00 *	Fear > anger, sadness, disgust, happiness > sadness, disgust, happiness, surprise
	Mean	9.045	0.00 *	Fear > anger, sadness, disgust, happiness > sadness, disgust, happiness, surprise

* The significance level was at *p* < 0.05.

**Table 4:** Results of classifier model for fear, anger, and surprise.

Classifier	AUC	CA	F1	Precision	Recall
K-nearest neighbor (KNN)	0.675	0.500	0.507	0.574	0.500
Decision Tree (DT)	0.760	0.654	0.653	0.653	0.654
Radom Forest (RF)	0.832	0.700	0.702	0.704	0.700
Logistic Regression (LR)	0.871	0.761	0.761	0.761	0.761

**Table 5:** Confusion Matrix on the logistic regression classifier for fear, anger, and surprise.

	Predicted	Anger	Fear	Surprise
True				
Anger		60.0%	40.0%	0.0%
Fear		36.7%	63.3%	0.0%
Surprise		0.0%	0.0%	100.0%

## Data Availability

The data used to support the findings of this study are available from the corresponding author upon request.
